# Unusual localization of bleeding under acenocoumarol: Spinal subdural hematoma

**DOI:** 10.1016/j.ijscr.2019.04.053

**Published:** 2019-05-10

**Authors:** Ismail Aissa, Abdelghafour Elkoundi, Rabi Andalousi, Aziz Benakrout, Abdelatif Chlouchi, Mohamed Moutaoukil, Jawad Laaguili, Mustapha Bensghir, Hicham Balkhi, Salim Jaafar Lalaoui

**Affiliations:** a Department of Anesthesiology and Intensive Care, Military Hospital Mohammed V, Faculty of Medicine and Pharmacy of Rabat, Mohammed V University, Rabat, Morocco; b Department of Neurosurgery, Military Hospital Mohammed V, Faculty of Medicine and Pharmacy of Rabat, Mohammed V University, Rabat, Morocco

**Keywords:** Spinal subdural hematoma, Acenocoumarol, Spinal cord compression

## Abstract

**Background:**

The spinal subdural hematoma (SSH) is an extremely rare entity which represents only 4.1% of all spinal hematomas. It needs accurate diagnosis and rapid intervention because of the major neurological risk induced by spinal compression. Several etiologies have been reported: anticoagulant treatments, haematological disorders, arterio-venous malformation, repeated attempts at lumbar punctures and tumors. We report the case of an 82-year-old patient under acenocoumarol for atrial fibrillation who presented with paraplegia secondary to SSH.

**Case report:**

An 82-year-old patient with a history of ischemic heart disease and atrial fibrillation under acenocoumarol was admitted to emergency department with sudden onset of paraplegia and intense back pain associated with urinary incontinence and anal sphincter disorder. On examination his lower limb power was MRC grade 0 out of 5 in all ranges of movement bilaterally and a complete bilateral anesthesia reaching the T12 dermatome was noted. Biological test results showed an International Normalized Ratio at 10. Magnetic resonance imaging revealed a posteriorly located spinal hematoma at T12 level, measuring 36 mm with spinal cord compression. After correction of hemostasis disorders the patient was admitted to the operating room for a T11-L1 laminectomy with evacuation of the subdural hematoma. Muscle power showed a gradual improvement in the lower limbs estimated at 3/5 with regression of sphincter disorders but unfortunately a sequellar sensory impairment persisted.

**Conclusion:**

SSH is a rare situation of acenocoumarol bleeding incident, it should be evoked in any patient treated by this molecule with signs of spinal cord compression.

## Highlights

• The spinal subdural hematoma is a rare situation which should be evoked in any patient treated by vitamin K antagonists with signs of spinal cord compression.
• Magnetic resonance imaging is the imaging exam of choice to establish the diagnosis.
• Rapid correction of bleeding disorders is required.
• Sometimes, emergent surgical evacuation of the hematoma is the only therapeutic option to ensure optimal neurological prognosis.
• The procedures for resuming anticoagulation should be subject to a multidisciplinary consultation.

## 1 Introduction

First described by Shiller et al. [1], the spinal subdural hematoma (SSH) is an extremely rare entity which represents only 4.1% of all spinal hematomas [2]. It needs accurate diagnosis and rapid intervention because of the major neurological risk induced by spinal compression. Several etiologies have been reported: haematological disorders, arterio-venous malformation, repeated attempts at lumbar punctures and tumors [[3], [4], [5], [6]].

We report the case of an 82-year-old patient under acenocoumarol for atrial fibrillation who presented with paraplegia secondary to SSH. This work has been reported in line with the SCARE criteria [7].

## 2 Case report

An 82-year-old patient with a history of diabetes mellitus, hypertension, ischemic heart disease and atrial fibrillation, was admitted to emergency department with sudden onset of paraplegia and intense back pain associated with urinary incontinence and anal sphincter disorder.

His regular medications were acenocoumarol 2 mg / day, ramipril, bisoprolol, furosemide, metformin and simvastatin. No trauma occurred in the days preceding his neurologic symptoms.

On examination, he was conscious and well oriented, lethargic, and afebrile. His blood pressure was 150/80 mmHg, and heart rate 120 beats / min. His lower limb power was MRC grade 0 out of 5 in all ranges of movement bilaterally. Osteo-tendinous reflexes were abolished, and a complete bilateral anesthesia reaching the T12 dermatome was noted. The anal sphincter tone was also reduced.

**Fig. 1:**
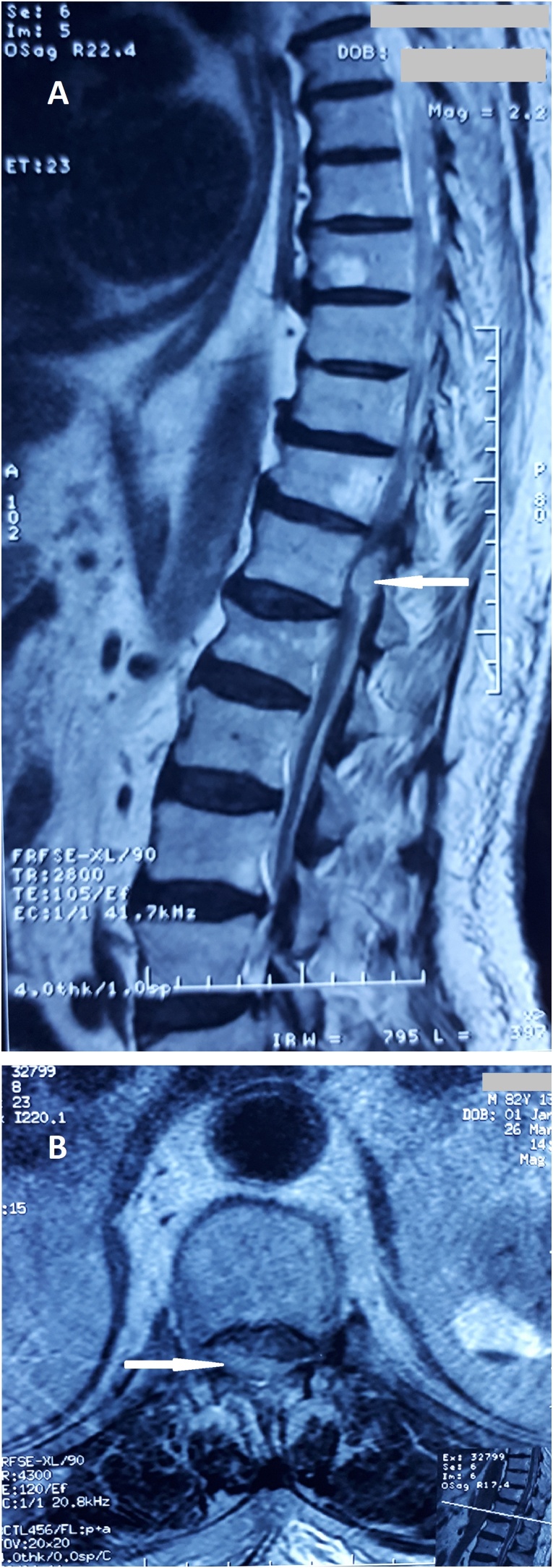
preoperative images from (A). sagittal T2-weighted magnetic resonance imaging (MRI) scan, (B). axial T2-weighted MRI revealed a spinal subdural hematoma at T12 (arrows) compressing the myelum from behind.

Biological test results showed an International Normalized Ratio (INR) at 10, a normal level of platelets, and a renal insufficiency (urea 1.71 g / l, creatinine 27 mg / l). Magnetic resonance imaging (MRI) revealed a posteriorly located spinal hematoma at T12 level, measuring 36 mm with spinal cord compression (Fig. 1). There was no tumor or underlying vascular malformation.

A pericardial effusion was individualized on Trans-thoracic echocardiography effusion (approximately 300 ml) along with a septal hypokinesis and left ventricular hypertrophy.

**Fig. 2:**
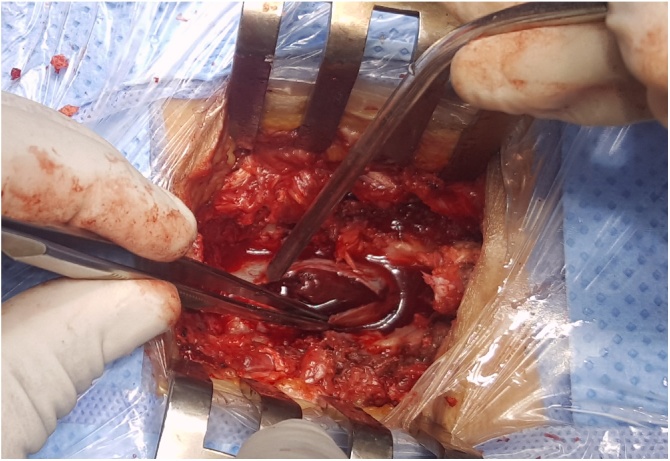
Intraoperative photograph showing the spinal subdural hematoma.

The patient received 10 mg of vitamin K intravenously and 10 units of fresh frozen plasma. After obtaining an INR of 1.4, the patient was admitted to the operating room for a T11-L1 laminectomy with evacuation of the subdural hematoma (Fig. 2). The operative findings did not reveal any arteriovenous or neoplastic malformation. At the end of the surgery, the patient was transferred to the intensive care unit and then to neurosurgery ward.

After consultation with the surgeon and the cardiologist, the anticoagulant treatment was interrupted for 6 days after the surgery and then reintroduced for 72 h with heparin sodium in continuous infusion (in order to obtain an activated partial thromboplastin time target between 2 and 3 times the control). Acenocoumarol was then started targeting an INR of 2.5 which authorized stopping heparin infusion.

Muscle power showed a gradual improvement in the lower limbs estimated at 3/5 with regression of sphincter disorders but unfortunately a sequellar sensory impairment persisted. Spinal MRI performed 3 weeks later showed complete resolution of the hematoma. The patient was then referred to the Physical Rehabilitation Department for additional care.

## 3 Discussion

The haemorrhagic events due to vitamin K antagonists (VKA) represent the leading cause of iatrogenic hospitalization in France (17,000 / year) and the third in the United Kingdom [8,9]. Clinical trials have shown that VKA increases the risk of major bleeding by 0.5% per year and the risk of intracranial hemorrhage by approximately 0.2% per year [10]. The effectiveness of this therapeutic class in the prevention of thromboembolic events has been demonstrated in many studies, however, their use requires regular biological monitoring.

Several risk factors have been associated with the risk of bleeding during VKA treatment; overdosage has been clearly identified in the literature as a major risk factor. In the context of atrial fibrillation, the risk of haemorrhage increases by a factor of 30 for an INR greater than 4 [11]. Age is also a major risk factor. The relative risk of intracranial hemorrhage was 2.5 (95% CI 2.3–9.4) in patients over 85 years old compared to patients aged between 70–74 years [10]. Instability of INR (poor adherence to therapy, taking drugs or foods interfering with AVK) was also reported as a risk factor [12].

Other factors have been implicated: history of gastrointestinal haemorrhage or stroke and some comorbidities (diabetes, renal failure, severe anemia, recent myocardial infarction, presence of neoplastic pathology) [13]. Some genetic factors have also been reported recently [14]. Our patient associated a lot of risk factors including INR at 10, advanced age, diabetes and kidney failure.

The localization of bleeding under VKA is most often gastrointestinal, urinary or cerebral. SSH is an atypical manifestation of VKA overdosage. Domenicucci et al. found that in 106 cases of non-traumatic SSH, 35% of the patients were under anticoagulant therapy [15]. Only a few number of them were on VKA. Warfarin is mostly associated with SSH, while acenocoumarol has been sparsely reported.

SSH is most often located in the thoracic region [16]. The etiopathogenic mechanisms involved in the constitution of SSH remains unclear [[17], [18], [19]]. In the subdural spinal space, there are no major blood vessels in contrast with the spinal epidural space or the intracranial subdural space [18]. A theory has been raised that hemorrhage originates in the more vascularized subarachnoid space, probably after high intra-abdominal or intrathoracic pressure, and then breaks through the very fragile arachnoid membrane in the subdural space where the hematoma is constituted. This theory seems unlikely in our patient in whom the surgical exploration did not find any break-in of this membrane. Morandi et al. suggest in a similar situation that the subdural hematoma could come from small broken vessels on the inner surface of the dura mater [17].

SSH can have a wide spectrum of presentation ranging from spinal pain radiating sometimes to limb or trunk to acute motor deficits depending upon severity, rapidity and level of compression [19]. Urinary or fecal incontinence can be observed. However, some cases were described with no motor or sensory deficits [20].

These symptoms must lead to the realization without delay of a spinal MRI which is considered the investigation of choice. Delimiting the dura mater and differentiating a subdural hematoma from an epidural hematoma can be difficult. The latter usually appears as a biconvex lesion with wide sagittal insertion base, while the SSH tends to be agglomerated and concave [17]. MRI also determines height extension, and the existence of underlying lesions. Some authors recommend the use of spinal angiography whenever it is available as it makes it possible to search for some possible arteriovenous fistulas, malformations or vascular aneurisms [19,21].

Treatment options includes a correction of bleeding disorders; VKA should be discontinued immediately upon suspicion. Rapid antagonism requires the administration of clotting factor concentrates or fresh frozen plasma, and administration of vitamin K [22]. The goal is to bring the INR to values below 1.5 as quickly as possible.

Patients with major deficits or with clinical and radiological aggravation (CT, MRI) should be treated urgently, even after long compression of the spinal cord. In such cases early decompression by laminectomy with evacuation of the hematoma is considered to be the best treatment [23].

Conservative treatment may be discussed in cases with minimal neurological deficits, early spontaneous recovery, or when the general condition of the patient is precarious [15]. The neurological prognosis of SSH is conditioned by several factors: the size of the hematoma, the induced medullary lesions, the extent of the initial deficit, the importance of the overdosage of VKA and especially the interval between the onset of symptoms and decompression [2].

The decision of whether and when to resume anticoagulation following a bleeding event under VKA is challenging and requires an assessment of associated risks and benefits. Apart from intracerebral hemorrhage, a 48–72 -h therapeutic window is recommended [24].

The prevention of haemorrhagic accidents under VKA remains crucial to avoid such a dramatic situation. It is primarily based on the continuous information and education of patients allowing better adherence to treatment. It has been clearly demonstrated that the management of these molecules by a specialized center in anticoagulation leads to a better therapeutic efficacy with a clear reduction in side effects [25]. Finally, several studies have identified that new oral anticoagulants were an alternative with a lower risk of bleeding in patients with atrial fibrillation [26,27].

## 4 Conclusion

SSH is a rare situation which should be evoked in any patient treated by VKA with signs of spinal cord compression. MRI is the imaging exam of choice to establish the diagnosis. Rapid correction of bleeding disorders is required. Sometimes, emergent surgical evacuation of the hematoma is the only therapeutic option to ensure optimal neurological prognosis. Finally, the procedures for resuming anticoagulation should be subject to a multidisciplinary consultation.

## Conflicts of interest

The authors declare no conflicts of interest associated with this manuscript.

## Sources of funding

None.

## Ethical approval

Ethical approval has been exempted by our institution.

## Consent

Written informed consent was obtained from the patient for publication of this case report and accompanying images. A copy of the written consent is available for review by the Editor-in-Chief of this journal on request.

## Author contribution

All authors contributed the perioperative management and writing this paper. Study design and manuscript was performed by I. Aissa, and other authors read this manuscript and approved submission. M. Bensghir, H. Balkhi, SJ. Lalaoui were decided final decision for submission, other authors discussed this manuscript together.

## Registration of research studies

This in not human study.

## Guarantor

Dr I. Aissa is the Guarantor of this report and has full responsibility to it.

## Provenance and peer review

Not commissioned, externally peer-reviewed
